# Protist species richness and soil microbiome complexity increase towards climax vegetation in the Brazilian Cerrado

**DOI:** 10.1038/s42003-018-0129-0

**Published:** 2018-09-06

**Authors:** Ademir Sergio Ferreira de Araujo, Lucas William Mendes, Leandro Nascimento Lemos, Jadson Emanuel Lopes Antunes, Jose Evando Aguiar Beserra, Maria do Carmo Catanho Pereira de Lyra, Marcia do Vale Barreto Figueiredo, Ângela Celis de Almeida Lopes, Regina Lucia Ferreira Gomes, Walderly Melgaço Bezerra, Vania Maria Maciel Melo, Fabio Fernando de Araujo, Stefan Geisen

**Affiliations:** 1 Agricultural Science Center, Federal University of Piauí, 64049-550 Teresina, PI Brazil; 2 Cell and Molecular Biology Laboratory, Center for Nuclear Energy in Agriculture CENA, University of Sao Paulo USP, 13416-000 Piracicaba, SP Brazil; 3 Genome Laboratory Agronomic Institute of Pernambuco, 50761-000 Recife, PE Brazil; 4 Laboratory of Microbial Ecology and Biotechnology, Lembiotech, Federal University of Ceara, 60020-181 Fortaleza, CE Brazil; 5 Universidade do Oeste Paulista, 19050-920 Presidente Prudente, SP Brazil; 6 Department of Terrestrial Ecology, Netherlands Institute of Ecology NIOO-KNAW, 6708 PB Wageningen, The Netherlands

## Abstract

Biodiversity underlies ecosystem functioning. While aboveground biodiversity is often well studied, the belowground microbiome, in particular protists, remains largely unknown. Indeed, holistic insights into soil microbiome structures in natural soils, especially in hyperdiverse biomes such as the Brazilian Cerrado, remain unexplored. Here, we study the soil microbiome across four major vegetation zones of the Cerrado, ranging from grass-dominated to tree-dominated vegetation with a focus on protists. We show that protist taxon richness increases towards the tree-dominated climax vegetation. Early successional habitats consisting of primary grass vegetation host most potential plant pathogens and least animal parasites. Using network analyses combining protist with prokaryotic and fungal sequences, we show that microbiome complexity increases towards climax vegetation. Together, this suggests that protists are key microbiome components and that vegetation succession towards climax vegetation is stimulated by higher loads of animal and plant pathogens. At the same time, an increase in microbiome complexity towards climax vegetation might enhance system stability.

## Abstract (web summary)

Araujo et al. investigate the soil microbiome across four major vegetation zones of the Brazilian Cerrado and find that protist taxon richness increases towards the tree-dominated climax vegetation. Their findings suggest that increased microbiome complexity might enhance system stability towards climax vegetation.

## Introduction

Biodiversity is of key importance for ecosystem functioning^1^. However, biodiversity is generally declining, particularly due to anthropogenic processes, including overexploitation, agriculture, invasive species and climate change^2^. Tropical rainforests are often studied as hotspots of biodiversity, whereas other biodiversity hotspots receive little attention. Among those are savannas, which host a unique flora and fauna, including the largest terrestrial mammals^3^.

With a size comparable to Europe, the Brazilian Cerrado is the largest and most taxon-rich savanna in the world^4,5^. This biome is composed of four main zones based on vegetation composition: a grass, grass and shrub, shrub and tree and tree-dominated zones^4,6^. Accordingly, these zones differ not only in their plant composition, but also in diversity, richness and density, which increases towards tree-dominated climax vegetation (Supplementary Table 1). A large part of this aboveground biodiversity is threatened^3,7^, mainly due to the increase in agriculture^8^. Therefore, efforts need to be expanded to protect this diversity. Biodiversity losses might be particularly prevalent and important among less studied groups of life, particularly soil biota, but we lack even a basic understanding of the diversity, connectedness and ecological importance of virtually all soil biota^9,10^. Increasing knowledge on soil biodiversity is obtained from more agriculture dominated regions, especially Europe and North America^11,12^, while the soil biodiversity in southern hemisphere soils, such as the tropical Cerrado, is much less studied. Only recently have microbial groups, including bacteria^13–15^, archaea^16^ and fungi^17,18^, been investigated in the Cerrado. These studies have revealed differences in the community composition of microbial groups between the four vegetation zones with diversity increasing from primary grass to climax tree vegetation. However, the community structure and distribution of the other microbial group, namely protists, remain unstudied in the Brazilian Cerrado as in most other soils.

This is surprising considering that protists constitute the vast majority of eukaryotes^19,20^ and are functionally versatile^21^. In soils, protists are the main consumers of bacteria and fungi and thereby drive elemental cycling^22^. Phototrophic soil protists fix carbon^23^. Diverse apicomplexan and other groups of soil protists are animal parasites^24^ and might contribute to animal diversity^25^. Furthermore, many protists, including fungal-like, yet phylogenetically unrelated, oomycetes and plasmodiophorids, are plant pathogens^26^. This functional diversity of protists might provide information on soil and ecosystem states, as has recently been suggested for plant pathogenic protists in agricultural settings^27^, although this indicative value of assigning taxa to potential functions has rarely been investigated for protists. Furthermore, we are only beginning to understand environmental drivers of protist communities in soils and observing that protist communities are differently structured than their bacterial and fungal counterparts. Protist communities seem mostly to be affected by soil moisture^28,29^ and type of plants^30^, but other abiotic factors, such as pH^31^ and litter chemistry^32^, contribute to shaping protist communities.

The lack of an understanding of the taxonomic and functional diversity of soil protists prevents a comprehensive understanding of the interconnectedness and potential function of entire soil microbiomes. Studies focusing on individual microbial groups are valuable as they provide in-depth knowledge on potential abiotic and biotic parameters that shape their communities^11,12,29,33^. However, potential interactions within the microbiome, including bacteria, archaea, fungi and protists, need to be considered to obtain a complete understanding of microbial community composition in soils. Otherwise, interactions within the soil food web such as top-down community controls remain masked even though that can be the main determinants of the performance and structure of communities^20,34,35^.

In this study, we aim to investigate the diversity of protists along four different vegetation zones in the Brazilian Cerrado using high-throughput sequencing of the hypervariable V9 region of the 18S rRNA gene^36^. We tested the hypotheses that the taxonomic and functional diversity of protists increases along a vegetation gradient of four zones ranging from primary grass to tree-dominated climax vegetation, and that microbiome connectedness increases towards climax vegetation in the Cerrado that might explain more stable (resistant and resilient) climax ecosystems^37,38^. To determine the microbiome structure, we combined sequence data of bacteria, archaea, fungi and protists. We show that protist taxon richness is lowest in primary grass vegetation with highest leads of plant pathogenic and animal parasitic taxa, while most protist taxa are found in climax tree vegetation. Network analyses reveal that protists are well embedded in the microbiomes, especially towards climax stage, suggesting their importance in microbiome functioning. Furthermore, an increased complexity of microbiome networks might support their stability.

## Results

### Abiotic soil analyses

Abiotic soil parameters in the four vegetation zones differed most between primary grass and climax tree zones. Increases in soil nitrogen (6.0-fold increase), soil water content (4.4×), total organic carbon (3.5×), potassium (2.7×), cation exchange capacity (2.1×) and available phosphorus (P, 1.4×) were observed in the tree relative to the grass zone (Supplementary Table 2). Also, soil pH was 0.6 higher, while temperature was 4.3 °C lower in the tree than in the grass vegetation zones. Grass-shrub and shrub-tree vegetation zones showed mostly similar abiotic values that were generally in between those observed in the grass and the tree zone (Supplementary Table 2).

### Eukaryotic community composition

The eukaryotic community profiling using 18S rRNA gene sequencing generated 2,570,000 sequences, with 1,835,000 sequences remaining for downstream analysis after quality filtering, which were assigned into 2513 operational taxonomic units (OTUs). Most OTUs were assigned to fungi (41.5%), protists (30.2%) and animals (25%), with low OTU representation of plant (3.3%) OTUs. In order to test the reliability of all results, we processed the sequences using SWARM. Although the number of OTUs increased (4940), taxonomic annotations of these SWARMs were nearly the same as for the OTUs based on 97% similarity. In short, most eukaryotic OTUs were assigned to fungi (41%), followed by animals (23%) (including nematodes 4%), protists (32%) and plants (4%). While we processed all data simultaneously based on OTUs and SWARMs, we focus here the OTUs based on 97% similarity for further analysis, as most studies use this conservative closed-reference OTU-picking approach. We report, however, analyses on the SWARMs in the supplementary information and discrepancies between both in the main text.

**Fig. 1:**
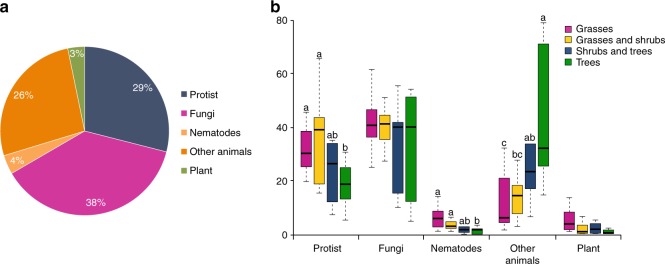
The composition of eukaryotic communities based on 18S rRNA gene. **a** General abundance of sequences affiliated to eukaryote groups across all Cerrado vegetation zones. **b** Comparison of eukaryotic abundances between different vegetation zones. Different lowercase letters refer to significant differences between the vegetation zones (White’s non-parametric *t*-test, *P* < 0.05). ‘Other animals’ include Annelida, Arthropoda, Cnidaria, Mollusca, Gastrotricha, Platyhelmintes, Porifera, Rotifera and Tardigrada

In terms of read abundance, most eukaryotic reads were assigned to fungi (38%), followed by animals (30%) (including nematodes 4%), protists (29%) and plants (3%) (Fig. 1a). The relative abundance of fungi did not differ between the zones (Fig. 1b). Protists and nematodes decreased towards the tree zone (*P* < 0.05), while ‘other animals’ increased (*P* < 0.05; Fig. 1b). These patterns were identical for the analyses of the SWARMs with the exception that the decrease of protists towards the tree zone was not significant (Supplementary Fig. 2B).

### Protist community patterns along the vegetation zones

**Fig. 2:**
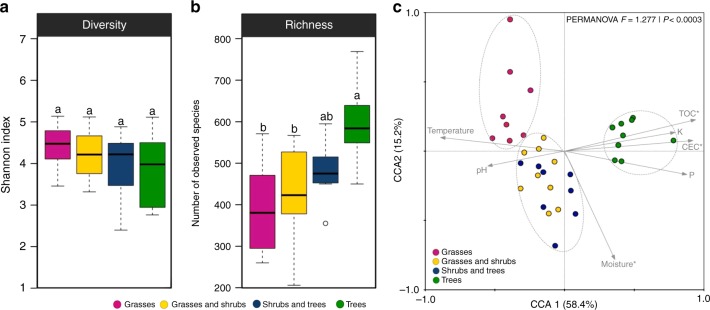
Diversity and structure of protist communities in soils from a vegetation gradient of the Brazilian Cerrado. Taxonomic diversity (**a**) and richness (**b**) are based on OTU level affiliated to PR^2^ at 97% similarity. Error bars represent the standard deviation of ten independent replicates. Different lowercase letters refer to significant differences between treatments based on Tukey’s HSD test (*P* < 0.05). **c** Canonical correspondence analysis (CCA) of protist community patterns and soil characteristics. Arrows indicate correlation between environmental parameters and protist profile. The significance of these correlations was evaluated via the Monte Carlo permutation test and is indicated by * (*P* < 0.05). Significant clusters (PERMANOVA, *P* < 0.05) are indicated by dashed lines in the graph

Protist alpha diversity was similar in all four zones (Fig. 2a, Supplementary Fig. 3A), while taxon richness was higher in tree (on average 597 OTUs) compared to grass-shrub (on average 432 OTUs) and grass zones (on average 397 OTUs; *P* < 0.05; Fig. 2b). However, no significant changes in protist alpha diversity between the zones were observed based on SWARMs (Supplementary Fig. 3B). The tree zone hosted most OTUs that were specific for a single zone (18.8% of all OTUs). In line with this observation, more than half of all obtained OTUs were found in the tree zone (50.7%), while the fewest were observed in the grass zone (41.0%, Supplementary Fig. 1). Yet, a substantial percentage of OTUs (13.3%) were shared between all four zones.

The structure of protist communities differed between the vegetation zones, which could be attributed to changes in abiotic parameters (Fig. 2c; Supplementary Fig. 3C). The first two axes of the graph explained more than 70% of the data variation and clustered the samples in three main groups, grass zone samples, grass-tree and shrub-tree zone samples and tree zone samples, as confirmed by PERMANOVA (*F* = 1.3, *P* < 0.001). Increased total organic carbon, potassium, cation exchange capacity and available phosphorus structured protist communities in the tree zone, while increasing temperature and reduced soil moisture determined protist communities in the grass zone (Fig. 2c; Supplementary Fig. 3C). Protist communities in the intermediate, overlapping zones, were structured by higher pH and soil moisture (Fig. 2c; Supplementary Fig. 3C). Also, a canonical correspondence analysis (CCA) followed by Monte Carlo analysis indicated that soil moisture (*F* = 2.26, *P* < 0.001), total organic carbon (*F* = 2.55, *P* < 0.001) and cation exchange capacity (*F* = 1.88, *P* < 0.001) were the most important abiotic factors that correlated with general community structure of soil protists.

### Protist taxonomic and functional group composition along the vegetation zones

**Fig. 3:**
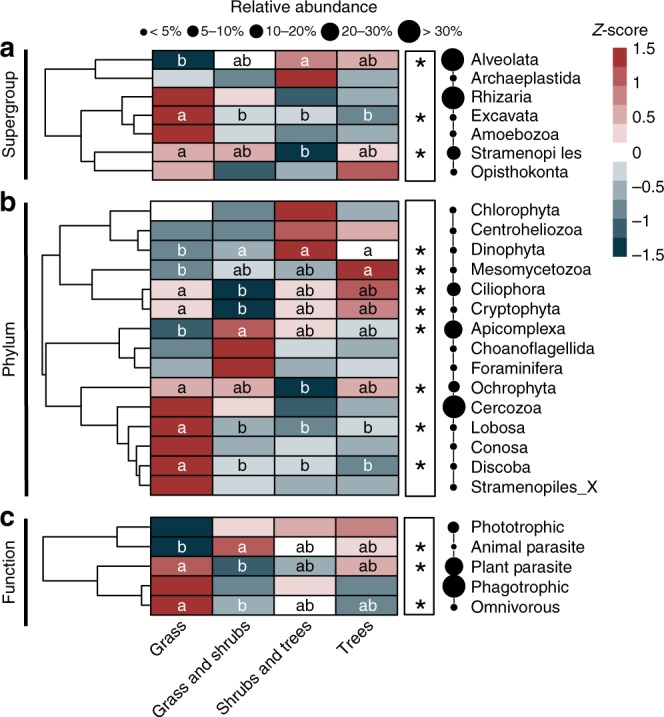
Heatmaps showing the differential abundance of protist supergroup (**a**), phyla (**b**), and functional categories (**c**) in soils from the vegetation gradient. The colour key relates the heatmap colours to the standard score (*z*-score), i.e. the deviation from row mean in units of standard deviation above or below the mean. Asterisks indicate significantly different group abundances (White’s non-parametric *t*-test, *P* < 0.05), which is illustrated by different lowercase letters inside the boxes. Circles are proportional to the relative abundance of each group in all samples. Only phyla with average abundance > 0.01% were included in the figure

Alveolates (44.2% of all protist reads, 35.2% of all OTUs) and Rhizaria (reads: 40.9%, OTUs: 36.2%) dominated the protist community, followed by Stramenopiles (reads: 8.4%, OTUs: 11.9%), Excavata (reads: 2.6%, OTUs: 5.3%), Amoebozoa (reads: 1.6%, OTUs: 5.3%) and Archaeplastida (reads: 1.0%, OTUs: 2.3%) (Fig. 3a). Alveolates were particularly abundant in the shrub tree compared with the grass zone (*P* < 0.05), while the opposite was observed for Stramenopiles (*P* < 0.05; Fig. 3a). Excavates were highest in the grass compared to all other zones (*P* < 0.05; Fig. 3a).

Cercozoa (Rhizaria) was the most common and OTU-rich phylum (reads: 40.9%, OTUs: 35.8%), with no difference in reads or OTUs between vegetation zones (Fig. 3b). Apicomplexa (Alveolata) represented on average 21.2% of all protist reads (7.7% of all OTUs), being twofold more abundant in the grass-shrub than in the grass zone (*P* < 0.05). The opposite pattern with a 1.5-fold greater abundance in the grass than in the grass-shrub zone was observed for the Ciliophora (*P* < 0.05), a diverse group containing mostly ciliates (reads: 18.3%, OTUs: 20.3%; Fig. 3b). Also, less abundant phyla differed between the zones such as Dinophytes being 20-fold less abundant in the grass than in other zones, parasitic Mesomycetozoa being 27-fold enriched in tree zone compared with the grass, Cryptophytes being sixfold more abundant in the grass than in the grass-shrub zone, Ochrophytes being twofold higher in grass than in the shrub-tree zone, while the Lobosa and Discoba were higher in grass than in all other zones (1.5 and 2-fold, respectively). All effects are summarised in Fig. 3b.

Based on functional groups, plant parasites and omnivores were more abundant (fourfold and twofold, respectively) in the grass than in the grass-shrub zone (*P* < 0.05; Fig. 3c). In contrast, the relative abundance of animal parasites was highest in the grass shrub (32% of the reads) and lowest in the grass zone (17%) (*P* < 0.05, Fig. 3c). Other functional groups were not significantly different between the four vegetation zones (Fig. 3c).

### Disentangling the microbiome

**Fig. 4:**
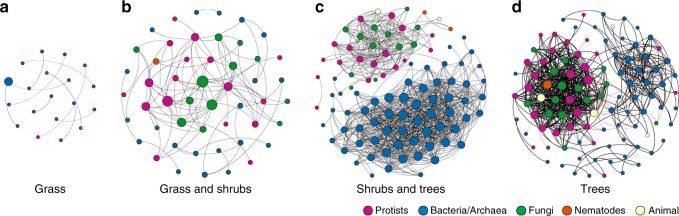
Network co-occurrence analysis of all microbiome eukaryotes and prokaryotes in soils from the vegetation gradient. A connection stands for SparCC correlation with magnitude of > 0.9 (positive correlation – black edges) or < −0.9 (negative correlation – red edges) and statistically significant (*P* < 0.001). Each node represents different prokaryotic or eukaryotic families, and the size of the node is proportional to the number of connections (degree)

Co-occurrence network analyses including soil bacteria, archaea, fungi, protists and animals showed that the network complexity (here defined by average changes in network properties, focusing particularly on nodes, edges and community hubs; Table 1) increased from the grass to the tree zone. The grass zone was least complex (19 nodes; 10 edges; nine community hubs; Fig. 4a), followed by the grass-shrub (50 nodes; 98 edges; eight communities; Fig. 4b) and the shrub-tree zone (97 nodes; 929 edges; five communities; Fig. 4c), with the climax tree zone being most complex (101 nodes; 606 edges; 16 communities; Fig. 4d; Table 1). The tree zone could also be distinguished from the shrub-tree zone by more complex correlations between all microbiome members, such as between fungi-protists (25.9% of all correlations), prokaryote-prokaryote (24.9%), protist-protist (20.6%), fungi-fungi (9.9%) and protist-animal (8.1%), while prokaryote-prokaryote interactions by far dominated interactions in the shrub-tree zone (80.4%, detailed information in Supplementary Information T1). Only the tree zone showed negative correlations between nodes (4.6% of all edges; Table 1, Supplementary Information T1) that were all formed between bacteria and non-bacterial taxa. These patterns of increased microbiome connectedness from grass towards climax tree zone based on family level were confirmed by OTU-level analyses (Supplementary Information T2).

**Table 1:** Correlations and topological properties of the networks

Network properties	Grass	Grass and shrubs	Shrubs and trees	Trees
Number of nodes^a^	19	50	97	101
Number of edges^b^	10	98	929	606
Positive edges^c^	10	98	929	578
Negative edges^d^	0	0	0	28
Modularity^e^	0.88	0.46	0.38	0.43
Number of communities^f^	9	8	5	16
Network diameter^g^	1	6	6	6
Average path length^h^	1	2.42	1.89	2.54
Average degree^i^	0.526	3.92	19.15	12
Average clustering coefficient^j^	0	0.54	0.74	0.72

^a^Microbial taxon (at family level) with at least one significant (*P* < 0.01) and strong (SparCC > 0.9 or < −0.9) correlation

^b^Number of connections/correlations obtained by SparCC analysis

^c^SparCC positive correlation ( > 0.9 with *P* < 0.01)

^d^SparCC negative correlation ( < −0.9 with *P* < 0.01)

^e^The capability of the nodes to form highly connected communities, that is, a structure with high density of between nodes connections (inferred by Gephi)

^f^A community is defined as a group of nodes densely connected internally (Gephi)

^g^The longest distance between nodes in the network, measured in number of edges (Gephi)

^h^Average network distance between all pair of nodes or the average length of all edges in the network (Gephi)

^i^The average number of connections per node in the network, that is, the node connectivity (Gephi)

^j^How nodes are embedded in their neighbourhood and the degree to which they tend to cluster together (Gephi)

## Discussion

This integrative microbiome study in natural soils which not only includes bacteria, archaea, fungi and protists, but also animals shows that complex communities spanning the entire microbiome highlights the importance of studying the entire microbiome to understand mechanisms that might underlie ecosystem functioning. The focus was on unstudied protists in the Brazilian Cerrado, which formed distinct communities in the four different vegetation zones and were particularly different between the grass and the tree zone. Similarly, protist taxon richness increased towards the climax tree zone vegetation. This is in line with patterns observed for other microbiome components, i.e. bacteria^13,15,18^, archaea^16^ and fungi^17,18^. These patterns can be attributed to abiotic differences, such as lower fertility and soil moisture, but higher acidity and soil temperature, in the grass compared to the tree zone. Abiotic differences were shown to drive above-ground plant community-dynamics across the Cerrado^39,40^, and therefore likely also belowground communities^13,16,17^. In line with other studies, we show that a combination of multiple abiotic factors drive protist communities, with soil moisture being the principle factor^28,29^.

The observed changes in species richness and community structure were not, however, mirrored in diversity analyses, as protist diversity was similar across zones. Changes in the relative abundances of different taxonomic groups of protists varied and no striking differences especially between the zones that differed mostly in the overall community structure and taxonomic richness (the grass and tree zone) were detectable. This shows that community analyses have to go beyond diversity to better understand underlying ecological patterns^41^. Furthermore, these results might imply that the dominant higher-trophic level organisms have broader niches such as being able to use a wider food resource spectrum than their bacterial and fungal prey. Similar relative abundances of phagotrophs, the dominant functional group of protists, were found in the four zones. Whereas phylogenetically distant phagotrophic protist taxa interact in a species-specific way with their microbial prey^42,43^, feeding differences among closely related protist predators and their prey might be less pronounced. This might explain why differences between phagotrophs might not have been found.

Alveolates and Rhizaria dominated the soil protist communities as in other studies^25,31,44^, but parasitic apicomplexans (Alveolata) were less abundant than in a recent report from Neotropical rainforests^25^. This can be explained by a lower diversity of potential arthropod hosts in savannas compared with rainforests^45^, which we confirm with our data showing profoundly increased relative abundances of animals, including arthropods, towards the tree vegetation zone.

It has to be noted that with the data obtained we are not able to differentiate causes and consequences of biotic changes in both protist communities and the vegetation, but assume that there are complex interactions between plants, abiotic factors and the soil biota. Plant species are not only known to change all parts of their microbiome through exudation, but also through changes in their abiotic surroundings^30,34,46^. These changes often translate to changes in soil biota and in turn to plant performance^47^. Thus, richness and composition of soil biodiversity often is key to affect plant performance. Protists are key for microbiome functioning^20^, and therefore likely govern plant soil feedbacks. Food web interactions including the microbial loop make nutrients bound in microbial biomass available for plant uptake, and thereby support plant performance^20,48^. In contrast, plant pathogens such as oomycete protists negatively affect plant performance^20^. Hence, soil organisms are at least to some extent involved in the transition towards climax vegetation. Plant herbivores have long been known to stimulate plant vegetation dynamics^49^. Recently, plant pathogenic fungi have been suggested to drive succession after agricultural land abandonment as they were enriched in early and decreased in later successional vegetation stages^50^. We also found highest abundances of plant pathogenic protists in the primary grass zones in the Cerrado, which might induce turnover to later successional stages. Therefore, complex interactions of abiotic and biotic (especially plants, but also animal hosts for parasites) components lead to taxonomic and functional changes in protist communities, and in turn, soil food webs in natural systems. These likely feedback to plant performance as illustrated by the soil organism-driven restoration of ecosystems^51^.

Protists were well embedded within soil microbiome networks, which confirms a previous study conducted in a pot experiment^27^. In line with our hypothesis, we show that the microbiome complexity as here defined by the average network properties (Table 1) increased from the grass towards the tree zone. This can be explained by higher system stability in more diverse climax systems as previously found for increased connectedness and nutrient uptake-efficiency over time after agricultural land abandonments^37^. Interestingly, while the overall microbiome complexity was highest in the climax tree zone, the network connections decreased compared with the shrub-tree zone. This suggests that system stability is not determined by the number of network links after a certain threshold, but rather the type of these links. This notion is supported by a profound increase of connections between bacteria and other microbes in the tree zone while the bacterial part of the microbiome was almost entirely disconnected in the shrub-tree zone. Furthermore, negative interactions only appeared between microbes in the tree zone and contributed little to all correlations. Generally, correlations in networks among soil organisms seem to be dominated by positive links and so mutualistic interactions therefore outnumber negative interactions^52^. A better interconnectedness of the entire microbiome and not within individual groups of microorganisms as well as negative interactions might contribute to system’s stability^38^. This increased stability is predominantly suggested to occur if these interactions are caused by complex predator-prey interactions^53^. While we cannot provide ultimate proof that this scenario is present in the sampled climax vegetation of the Cerrado, the fact that only free-living bacterial groups were negatively correlated with eukaryotes suggests that this is actually the case. Enhanced environmental niches, increases in substrate complexity along with the observed higher nutrient availability in the climax vegetation might promote longer and more stable food webs^53^, such as shown in a gradient of increased substrate complexity^34^.

Taken together we show that the protist community is well embedded within the microbiome, especially towards climax vegetation. Due to the functional versatility of its components, protists likely act as a driver of microbial prey communities as well as contributing to plant and animal diversity in soil systems. Our data further suggests that climax vegetation in natural successions might be dependent on a highly interconnected microbiome that controls the microbial community composition and, consequently, the system’s performance. This new information might help future efforts to create more resistant soil ecosystems^54^ and to speed up succession^51^.

## Methods

### Study area

This study was conducted within Sete Cidades National Park (PNSC; 04°02’-08’S and 41°40’-45’W), located in northeastern state of Piauí, Brazil. The park covers an area of 6221 ha. The climate is sub-humid moist, with a deficiency of precipitation and minor annual differences in temperature with an average temperature of 25 °C. The area has an annual average rainfall of 1558 mm, which primarily occurs in February, March and April.

We evaluated preserved sites (with 1000 m^2^ each) in the Cerrado, part of a long-term ecological program (PELD-CNPq) of the Brazilian government, spanning a gradient of Cerrado formations, including a grass, grass and shrub, shrub and tree, and tree-dominated zone (Table 1).

### Soil sampling and chemical analysis

Three transects were initiated in March (wet season) 2014 that spanned all four vegetation zones and were placed in a distance of 25 m from another. Three samples per zone were taken in a distance of 50 m from another and soil samples were collected at a depth of 0–20 cm in total, nine samples were taken at each vegetation zone resulting in a total of 36 biological replicates. All 36 soil samples were immediately stored in sealed plastic bags and transported on ice to the laboratory. Soil was sieved through a 2-mm screen and homogenised before a portion of soil samples was stored in bags and kept at −20 °C for DNA analysis, while another portion was air-dried for chemical analyses.

Soil chemical properties were determined and measured using standard laboratory protocols. Soil pH was determined in a 1:2.5 soil/water extract. Available P and exchangeable K were extracted using the Mehlich-1 extraction method and determined by colorimetry and photometry, respectively (Supplementary Table 2). Total organic carbon was determined by the wet combustion method using a mixture of potassium dichromate and sulphuric acid under heating^55^.

### DNA extraction and library preparation

Soil DNA was extracted from 0.5 g (total wet weight) of soil using the PowerLyzer PowerSoil DNA Isolation Kit (MoBIO Laboratories, Carlsbad, CA, USA), according to manufacturer’s instructions. DNA extraction was performed in triplicate for each soil sample. Measurements of DNA quality and quantity were determined using a Thermo Scientific NanoDrop 2000.

For taxonomical profiling of the protist communities, amplifications targeting the hypervariable V9 region of the 18S rRNA gene were performed using the primers 1391F together with 1510R^36^. First step amplification comprised 25 μL reactions containing 14.8 µL of nuclease-free water (Promega, Madison, WI, USA), 2.5 µL of 10X High Fidelity PCR Buffer (Invitrogen, Carlsbad, CA, USA), 1.0 µL of 50 mM MgSO4, 0.5 µL of each primer (10 µM concentration, 200 pM final concentration), 1.0 unit of Platinum Taq polymerase High Fidelity (Invitrogen, Carlsbad, CA, USA), and 4.0 µL of template DNA (30 ng). PCR conditions were: 94 °C for 4 min, followed by 25 cycles at 94 °C for 45 s, 57 °C for 60 s, and 72 °C for 90 s, with a final elongation at 72 °C for 10 min. A second indexing PCR was performed by adding a unique pair of Illumina Nextera XT indexes (Illumina, San Diego, CA) to each sample. Each 50 μL reaction contained 23.5 uL of nuclease-free water (Certified Nuclease-free, Promega, Madison, WI, USA), 5.0 uL of 10X High Fidelity PCR Buffer (Invitrogen, Carlsbad, CA, USA), 4.8 uL of 25 mM MgSO4, 1.5 uL of dNTP (10 mM each), 5.0 uL of each Nextera XT index (Illumina, San Diego, CA, USA), 1.0 unit of Platinum Taq polymerase High Fidelity (Invitrogen, Carlsbad, CA, USA), and 5.0 uL of each product from previous PCR. PCRs conditions were: 95 °C for 3 min followed by eight cycles at 95 °C for 30 s, 55 °C for 30 s, and 72 °C for 30 min, with a final elongation at 72 °C for 5 min. PCR products were cleaned using Agencourt AMPure XP – PCR purification beads (Beckman Coulter, Brea, CA, USA), according to manufacturer’s manual, and were then quantified using a dsDNA BR assay Kit (Invitrogen, Carlsbad, CA, USA) on a Qubit 2.0 fluorometer. Once quantified, different volumes of each library were pooled into a single tube such that each amplicon was represented equally. After quantification, the molarity of the pool was determined and diluted to 4 nM, denatured, and then diluted to a final concentration of 8.0 pM with a 20% PhiX spike. The paired-end sequencing was performed with Miseq Reagent Kit v2 (500 cycles, 2 × 250 bp) on an Illumina Miseq sequencer (Illumina, San Diego, USA) at the Unidade Multiusuário do Núcleo de Pesquisa e Desenvolvimento de Medicamentos (UM-NPDM; Federal University of Ceará).

### Sequencing data processing

Sequence data were processed using QIIME^56^ following the UPARSE standard pipeline^57^, according to the Brazilian Microbiome Project^58^ to produce the final OTU files. In short, reads were truncated to 150 bp and quality-filtered by setting the maximum expected error value of 1.0. Next, pre-filtered reads were dereplicated and singletons removed. Sequences were clustered into OTUs based on 97% sequence similarity. Samples were rarefied to 5000 reads. Taxonomy of the OTUs was assigned using the RDP Classifier^59^ with the PR^2^ database^60^. Sequences were submitted to the NCBI Sequence Read Archive under the number SRP136562.

### Data analyses

CCA was used to determine the correlation between the community structure of protist OTUs and soil physicochemical properties. All matrices were initially analysed using detrended correspondence analysis (DCA) in order to evaluate the gradient size of the taxon distribution, which indicated non-linear data distribution (length of gradient > 4), revealing that the best-fit model for the data was CCA. Forward selection (FS) and Monte Carlo permutation tests were applied with 1000 random permutations to verify the significance of soil chemical properties upon the microbial community structure. CCA plots were generated using Canoco 4.5 (Biometrics, Wageningen, The Netherlands). We used permutational multivariate analysis of variance (PERMANOVA)^61^ to test whether sample categories harboured significantly different community structures. Alpha diversity was calculated from an abundance matrix using Shannon’s index. PERMANOVA and alpha diversity indices were calculated with the software PAST3^62^. A Venn diagram was constructed to verify the proportion of groups exclusive and shared between samples using the webtool Venny 2.1^63^. To determine the statistical differences between the soil gradient, the Statistical Analysis of Metagenomics Profile v2.1.3 (STAMP) software was used^64^ and *P* values were calculated using the two-sided Welch’s test^65^, and correction was made using the Benjamini-Hochberg false discovery rate^66^.

In addition, co-occurrence network analyses were performed in order to assess the connectedness among the microbial communities from the four distinct vegetation zones in the Brazilian Cerrado. Non-random co-occurrence analyses were performed using SparCC, a tool capable of estimating correlation values from compositional data^67^. For this, data tables affiliated to family level, including 18S rRNA data (protists, fungi, nematodes and animal) and 16S rRNA data (bacteria^15^ and archaea^16^), were used in the analysis. We performed the analyses on family level as previously suggested to remove artefacts from inaccurate annotation of lower taxonomic levels and the discrepancy of OTUs to sometimes consist of multiple species, while sometimes species can contain multiple OTUs^68^. Nevertheless, we constructed a correlation matrix focusing on all eukaryotic OTUs and prokaryotic OTUs with relative abundance higher than 0.02% (that represented > 85% of all prokaryotic sequences) to assess the stability of the network architecture obtained by the cumulative analysis at the family level. For each network, *P* values were obtained by 99 permutations of random selections of the data table, subjected to the same analytical pipeline. SparCC correlations with a magnitude of > 0.9 or < −0.9 and statistically significant (*P* < 0.01) were included into network analysis ( > 0.6 or < −0.6 for supporting information to confirm stability of the network structure independent of the value of the correlation coefficient). The nodes in the reconstructed networks represent the taxonomic groups at family level, whereas the edges (that is, connections) correspond to a strong and significant correlation between nodes. The topology of the networks was calculated based on a set of measures, including numbers of nodes and edges, modularity, number of communities, average path length, network diameter, average degree and clustering coefficient^69,70^. Co-occurrence analyses were carried out using the Python module ‘SparCC’ and networks visualisation and properties measurements were calculated with the interactive platform Gephi^71^.

### Data Availability

Sequences generated during the current study are available in the NCBI Sequence Read Archive under the number SRP136562. Raw datasets that are not included in the manuscript or in the supplementary information are available from the corresponding author upon request.

## Electronic supplementary material

Supplementary Data 1

Supplementary Data 2

Description of Additional Supplementary Files

Supplementary Information
