# Supplementary material for: Protist species richness and soil microbiome complexity increase towards climax vegetation in the Brazilian Cerrado
Source: Commun Biol. 2018 Sep 6;1:135. doi: 10.1038/s42003-018-0129-0 (PMC6127325; doi:10.1038/s42003-018-0129-0)
Supplement: Supplementary file 3 — Description of Additional Supplementary Files [file 42003_2018_129_MOESM3_ESM.docx]

**Description of Additional Supplementary Files**

File Name: Supplementary Data 1

Description: SparCC correlations among prokaryotes, fungi and all eukaryotes at family level shown in separate sheets for each of the four vegetation zones. Significant (P < 0.01) correlations with correlation coefficients of R > 0.9 and R > 0.6 are shown.

File Name: Supplementary Data 2

Description: SparCC correlations among prokaryotes, fungi and all eukaryotes at OTU level. Significant (P < 0.01) correlations with correlation coefficients of R > 0.9 and R > 0.6 are shown in separate sheets.
