# Supplementary material for: Protist species richness and soil microbiome complexity increase towards climax vegetation in the Brazilian Cerrado
Source: Commun Biol. 2018 Sep 6;1:135. doi: 10.1038/s42003-018-0129-0 (PMC6127325; doi:10.1038/s42003-018-0129-0)
Supplement: Supplementary file 4 — Supplementary Information [file 42003_2018_129_MOESM4_ESM.pdf]

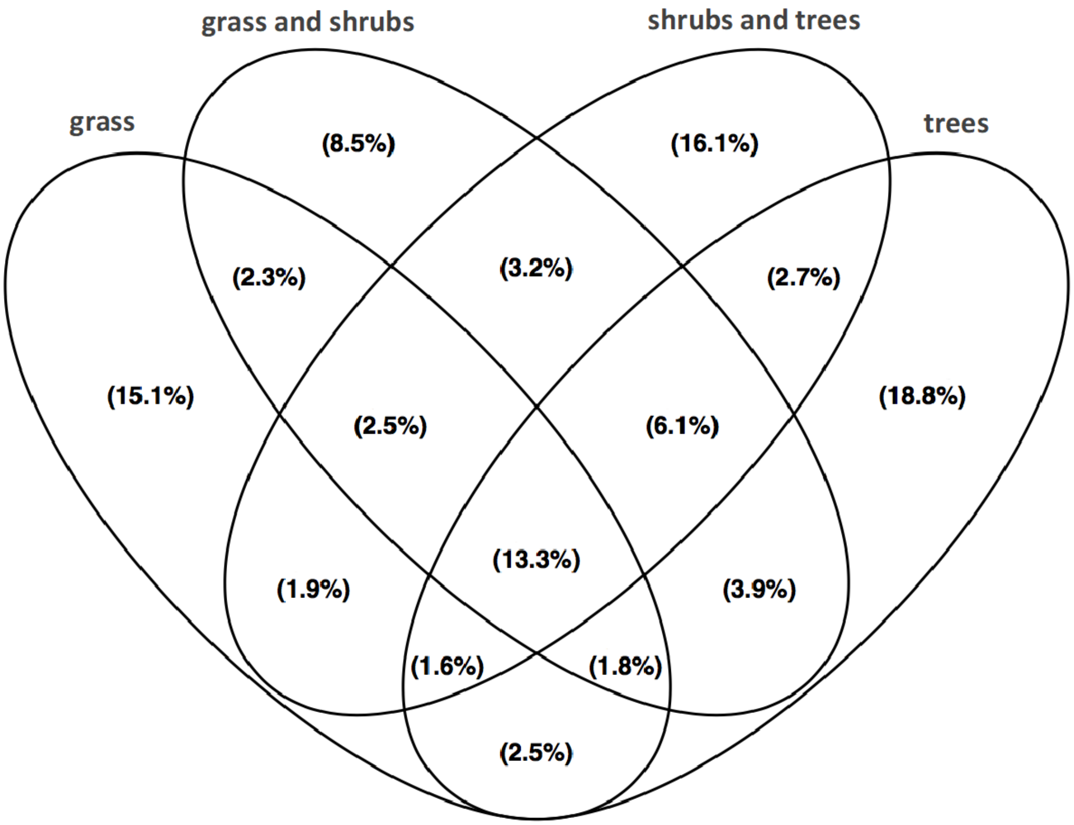

Supplementary Figure 1. Venn diagrams showing the number and proportion of unique and shared protist OTUs in soils from the four investigated vegetation zones.

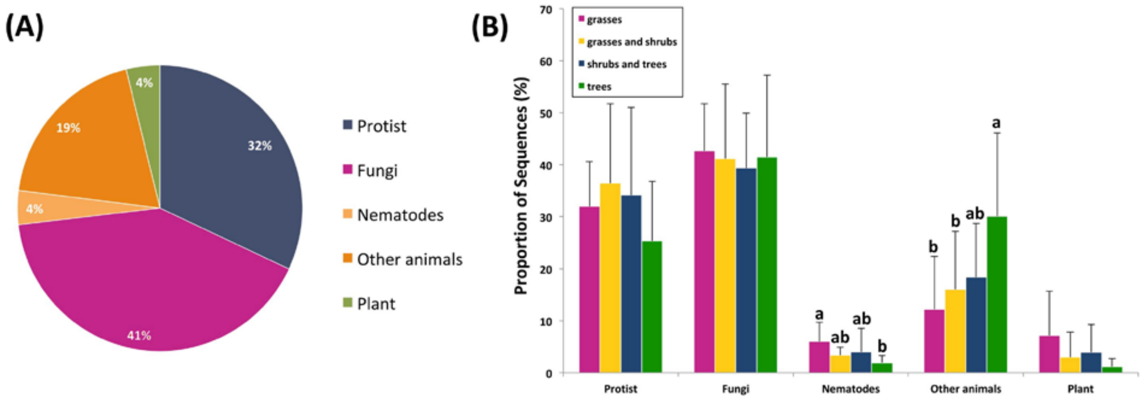

Supplementary Figure 2. The composition of eukaryotic communities based on 18S rRNA gene high throughput sequencing when OTUs where assigned using SWARM. **(A)** General abundance of sequences affiliated to eukaryote groups across all Cerrado vegetation zones. **(B)** Comparison of eukaryotic abundances between different vegetation zones. Different lower case letters refer to significant differences between the vegetation zones (White's non-parametric t-test, P = 0.045). 'Other animals' include Annelida, Arthropoda, Cnidaria, Mollusca, Gastrotricha, Platyhelmintes, Porifera, Rotifera and Tardigrada.

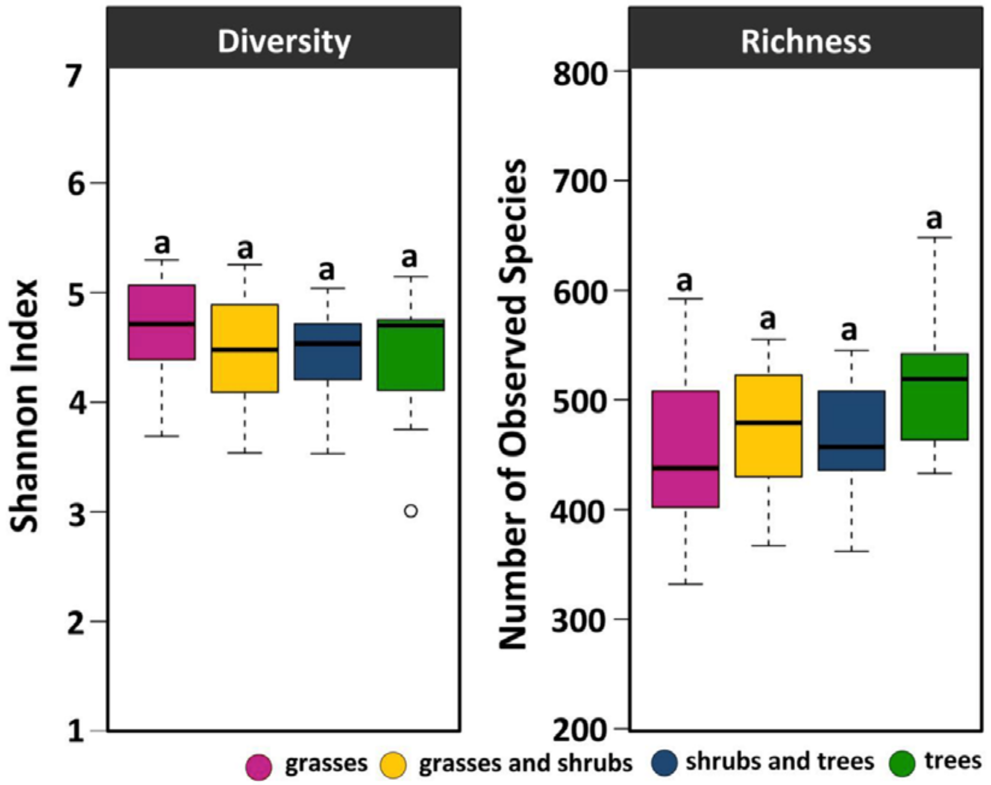

Supplementary Figure 3. Diversity and structure of protist communities in soils from a vegetation gradient of the Brazilian Cerrado when OTUs where assigned using SWARM. Taxonomic diversity **(A)** and richness **(B)** are based on OTU level affiliated to PR2 at 97% similarity. Error bars represent the standard deviation of ten independent replicates. Different lower case letters refer to significant differences between treatments based on Tukey's HSD test ($P < 0.05$). (C) Canonical Correspondence Analysis (CCA) of protist community patterns and soil characteristics. Arrows indicate correlation between environmental parameters and protist profile. The significance of these correlations was evaluated via the Monte Carlo permutation test and is indicated by * ($P < 0.05$). Significant clusters (PERMANOVA, $P < 0.05$) are indicated by dashed lines in the graph.

Supplementary Table 1. Vegetation diversity indices in the four vegetation zones in the Cerrado.

	Grass	Grass and shrub	Shrub and tree	Tree
Plant richness*	4.7	11	17	18
Plant diversity**	0.2	0.85	1.10	1.11
Plant density***	4.7	27.1	35.0	51.8
Vegetation****	a	b	c	d

* species/100 m^2; ** H/100 m^2; *** individual/100 m^2; **** species of plants present

[a] *Andropogon fastigiatus; Aristida longifolia; Eragrostis maypurensis*

[b]*Andropogon fastigiatus; Aristida longifolia; Terminalia fagifolia; Magonia pubescens; Hymenaea courbaril; Plathymenia reticulata; Qualea grandiflora; Combretum mellifluum; Lippia origanoides; Anacardium occidentale; Simarouba versicolor; Vatairea macrocarpa*

[c]*Aspidosperma discolor; Parkia platycephala; Terminalia fagifolia; Piptadenia moniliformis; Plathymenia reticulata; Qualea parviflora; Anacardium occidentale; Copaifera coriacea; Thiloa glaucocarpa; Casearia grandiflora.*

[d] *Aspidosperma multiflorum; Aspidosperma subincanum; Campomanesia aromática; Casearia lasiophylla; Casearia ulmifolia; Copaifera coriacea; Ephedranthus pisocarpus; Piptadenia moniliformis; Pterocarpus violaceus; Thiloa galucocarpa.*

Supplementary Table 2. Average of soil physicochemical properties across a gradient of Cerrado in Sete Cidades National Park, Northeast Brazil.

Site	Moisture (%)	Temperature (°C)	TOC g kg^{-1}	N g kg^{-1}	pH	P mg kg^{-1}	K cmolc kg^{-1}	CEC cmolc kg^{-1}
Grass	7.3^{c}	32.1^{c}	4.3^{c}	0.1^{c}	4.3^{c}	3.9^{c}	1.4^{b}	2.31^{b}
Grass and shrub	10.5^{b}	30.5^{b}	8.2^{b}	0.3^{b}	4.7^{a}	4.7^{b}	1.8^{b}	2.11^{b}
Shrub and tree	11.9^{b}	28.3^{a}	9.1^{b}	0.4^{b}	4.6^{ab}	4.5^{b}	1.6^{b}	2.35^{b}
Tree	31.8^{a}	27.8^{a}	15.2^{a}	0.6^{a}	4.9^{a}	5.3^{a}	3.8^{a}	4.91^{a}

TOC – total organic C; CEC – cation exchange capacity.

Values followed by the same letter within each column are not significantly different at the 5% level, as determined by Student's *t*-test.
